# Hyaluronan thiomer gel/matrix mediated healing of articular cartilage defects in New Zealand White rabbits—a pilot study

**DOI:** 10.1186/s40634-017-0089-1

**Published:** 2017-05-03

**Authors:** Christoph Bauer, Vivek Jeyakumar, Eugenia Niculescu-Morzsa, Daniela Kern, Stefan Nehrer

**Affiliations:** Center for Regenerative Medicine and Orthopedics, Department for Health Sciences and Biomedicine, Danube-University Krems, Dr.-Karl-Dorrek-Strasse 30, Krems, Austria

**Keywords:** Animal models, Chondrocytes, Articular cartilage, Polymers, Biomaterials

## Abstract

**Background:**

Articular cartilage defects are limited to their regenerative potential in human adults. Our current study evaluates tissue regeneration in a surgically induced empty defect site with hyaluronan thiomer as a provisional scaffold in a gel/matrix combination without cells on rabbit models to restore tissue formation.

**Methods:**

An osteochondral defect of 4 mm in diameter and 5 mm in depth was induced by mechanical drilling in the femoral center of the trochlea in 18 New Zealand White rabbits. Previously evaluated from an in vitro study hyaluronan thiomer matrix, and a hyaluronan thiomer gel was used to treat the defect. As a control, the defect was left untreated. During the whole study, rabbits were clinically examined and after 4 (*n* = 3) or 12 (*n* = 3) weeks, the rabbits were sacrificed. Joints were evaluated macroscopically (Brittberg score) and by histology (O’Driscoll score). Synovial cells from the synovial fluid smear were histopathologically evaluated.

**Results:**

The healing of the defects varied intra-group wise at the first observation period. After 12 weeks the results concerning the cartilage repair score were inhomogeneous within each group, while the macroscopic analysis was more homogenous. In the synovial fluid smear, the mean score of infiltrated synovial and non-synovial cells was slightly increased after 4 weeks and slightly decreased after 12 weeks in both the treatment groups in comparison to the untreated control.

**Conclusions:**

Taken together with results from the in vivo study indicated that implantation of hyaluronan thiomer as a combination of gel and matrix might enhance articular cartilage regeneration in an empty defect. Despite their benefits, the intrinsic healing capacity of New Zealand rabbits is a limitation for comparative test subject in pre-clinical models of cartilage defects.

## Background

The regeneration of articular cartilage has a limited potential as it is an avascular tissue and lacks access to progenitor cells (Solchaga et al. 2005; Aulin et al. 2013). Currently, many different biomaterials are under experimental investigation or are already used in clinical applications (Cook et al. 2016). These biomaterials are composed of natural polymers like collagen (Gille et al. 2010; Pascarella et al. 2010), agarose (Wang et al. 2011), hyaluronan (Solchaga et al. 2000; Kang et al. 2009), synthetic polymers (e.g. polylactic acid, polyglycolic acid) or a combination of both as composites. Hyaluronan (HA), a ubiquitous macromolecular polysaccharide of repeating units of N-acetyl-D-glucosamine and D-glucuronic acid is of great interest, as it is biocompatible, non-immunogenic and biodegradable (Necas et al. 2008). Currently, a diverse variety of HA-based biomaterials are available, but only a few are approved for clinical use (Albrecht et al. 2011). Non-crosslinked HA biomaterials degrade faster than crosslinked HA biomaterials under physiological conditions due to their high affinity to water and degradation by the enzyme hyaluronidase. HA biomaterials can be produced by chemical modification such as esterification of the carboxyl or hydroxyl groups by benzyl alcohol (HYAFF^TM^) (Benedetti et al. 1993). Auto cross-linking (non-covalent reactions), self-aggregation over hydrophobic interactions (Collins and Birkinshaw 2013; Dunkin and Lattermann 2013). Cross-Linking reactions can also be attained by carbodiimides, sulfides, aldehydes under acid, neutral or alkaline conditions or by the addition of synthetic linkers (Schanté et al. 2011). Alternatively, thiolated HA hydrogels offer better manipulation for mechanical properties and fabrication to defined shapes by either crosslinking with a thiol-reactive crosslinker or by oxidative disulfide formation (Wirostko et al. 2014). HA-based biomaterials are advantageous with biological properties that can bind to chondrocytes (Bauer et al. 2015) or mesenchymal stem cells (MSC’s) through the CD44 cell surface receptor (Jakobsen et al. 2010) and direct chondrogenic marker genes responsible for extracellular matrix production. HA scaffold investigated for one-stage cartilage repair combined with autologous bone marrow concentrates had superior clinical outcomes independent of the patient’s age or defect size post follow-up at 5 years (Gobbi and Whyte 2016).

Commonly used screening techniques for biomaterial based cartilage regeneration in pre-clinical small animal studies include by inducing a defect into the medial femoral condyle or the trochlear groove. Rabbits represent a species, which is very suitable to test new advanced biomaterials or new therapies as they can be handled easily, are relatively inexpensive and offer a good joint size for surgical procedures (Chu et al. 2010; Aulin et al. 2013). Larger animals, like e.g. sheep or goats, are relatively more expensive and reasonably considered to be used in later preclinical studies (Hurtig et al. 2011). The aim of the current pilot study is to examine the healing capacity of acellular cross-linked hyaluronan thiomer gel alone or gel + matrix scaffolds in surgically-induced chondral defects on femorotibial joints of New Zealand White rabbits.

## Methods

### Animals

The New Zealand White (NZW) rabbit is a suitable species for tolerability tests conventional to regulatory authorities and is entreated following the guideline ISO 10993–6. The study was conducted by the requirements of the Council Directive 86/609/EEC and subsequent amendments on the approximation of laws, regulations and administrative provisions regarding the protection of animals used for experimental and other scientific purposes or procedures. As no gender-specific differences are expected, only female animals were used in this study (Arzi et al. 2012). Eighteen NZW rabbits (8 months-old females, weight-range 4.3–5.5 kg) were purchased from S & K LAP Kft (Kartal, Hungary) in a good conventional health status. Before study initiation, the animals were accustomed to laboratory conditions for 2 weeks. A veterinarian on arrival examined the health status of the animals used in this study and before beginning the study. On the day of animal delivery, animals were allocated to the test groups and were marked individually by ear tags done by the supplier. Additionally, a waterproof ink was used to mark animals with their internal number on the inside of the ear. Room temperature was adjusted to 20 ± 3 °C, and the relative humidity was set at 30–70%. Artificial light was scheduled to give a cycle of 12 h light and 12 h dark (LD 12:12) with the light on at 6:30 a.m. Each rabbit was housed in a cage of stainless steel with the bottom grid. The animals were fed with a pellet diet and had access to tap water continuously.

### Preparation of the test items

#### HA matrix

Scaffolds made of cross-linked HA were produced and provided by Croma Pharma GmbH (Leobendorf, Austria). HA was derivatized with a linker containing a thiol group. Thiol-modified HA was dissolved in deionized water at a concentration of 1.5% (w/v) and the pH adjusted to 7.1–7.3 using NaOH. The solution was poured into 12-well plates (0.9–2 g per well for different hydrogel heights) and kept at room temperature for 5 h. Under these conditions, thiol groups slowly form disulfide bridges. The resulting cross-linked hydrogel was lyophilized to obtain HA matrix using the following parameters: The plates were frozen overnight in an −80 °C freezer, followed by the primary drying step for 24 h. The temperature of product footprint was −10–−15 °C, while the condenser temperature was −50–−55 °C. The vacuum applied was 0.05–0.1 mbar. The secondary drying step was for 2 h using the same parameters. The resulting HA matrix was punched to obtain sizes suitable for in vivo experiments.

#### HA gel

The 3.5% hyaluronan thiomer gel (HA gel) produced and provided by Croma Pharma GmbH (Leobendorf, Austria) in 80 mM phosphate buffer was diluted prior the surgical procedure with a buffer containing 0.06% H_2_O_2_ and 120 mM phosphate buffer. A ratio of 1:1 resulting in a final concentration of 1.75% HA gel, 0.03% H_2_O_2,_ and 100 mM phosphate buffer.

### Experimental design and surgical procedure

In all rabbits, a defect was created in the medial trochlear groove of the right knee. The 18 animals were divided into 3 groups where group 1 remained as an untreated control. The defect of group 2 was treated by filling with HA gel provided in a syringe. In group 3 the hyaluronan thiomer matrix was put into the defect, and the remaining unfilled space was filled up with hyaluronan thiomer gel so that the matrix (HA gel + matrix) will stay in the defect. After the observation periods (4 and 12 weeks) the rabbits were killed (Table 1).

**Table 1:** Experimental design—allocation of animals to treatment groups

					Implantation		
Group	Part	Animal no.	Treatment group name	Treatment	Knee	Injection volume gel [μl]	Observation period
1	II	101–103	Untreated control	-	right	-	12 weeks
	I	104–106					4 weeks
2	II	107–109	Gel	1.75% hyaluronan thiomer gel	right	approx. 60	12 weeks
	I	110–112					4 weeks
3	II	113–115	Gel + matrix	1.75% hyaluronan thiomer gel + lyophilized 1.5% hyaluronan thiomer matrix	right	approx. 20	12 weeks
	I	116–118					4 weeks

As a prophylaxis, all animals received a subcutaneous injection of an antibiotic (8.0 mg Cefovecin/kg b.w., Convenia®) approximately 2 h before surgery. Preoperatively, the rabbits were anesthetized with the combination of Medetomidine (0.3 mg/kg b.w., Cepetor®) and Ketamin (20.0 mg/kg b.w., Ketavet®) both administered intramuscularly. Analgesia was achieved with Carprofen (5.0 mg/kg b.w., Rimadyl®) given subcutaneously on the day of surgery and 6 days post-surgically. The animals received additional doses of Carprofen during the observation period when exhibited signs of pain.

The knee region was shaved and aseptically cleaned with an appropriate disinfection agent. The rabbits were covered with a sterile foil to keep them warm during surgery. Arthrotomy of the femorotibial joint was performed under aseptic conditions by an incision of the skin over the ligamentum patellae and an incision of the knee capsule. After dislocating the patella laterally, a chondral defect using a cordless screwdriver was drilled in the center of the trochlea. The defect was 4 mm in diameter, maximum 5 mm in depth and about 5 mm in the distance to the proximal trochlear ridge. Immediately after drilling the defect was rinsed with isotonic saline (0.9%). Then the HA gel was pipetted into the defect and let to polymerize for 3 min. For the animals of group 3 the lyophilized 1.5% hyaluronan thiomer matrix was placed into the chondral defect before it was filled up with HA gel. The defect of the untreated rabbits was rinsed before the knee capsule, and the skin region was sutured. The same closing procedure was performed in the treated rabbits. Aluminum Spray was used for wound closure. 30 min after surgery pain relief was reached by administrating Atipamezole (1.0 mg/kg b.w., i.m., Revertor®) to the animals.

Postoperatively observations were performed on day 1 of the study and monitored until the end of the observation period. Additionally, a cage side examination was performed daily on all animals. Particular attention was given to the surgery site and if the animal gained weight on the operated leg. At the end of both observation periods (4 and 12 weeks), the animals were sacrificed by an intravenous overdose of pentobarbiturate (150 mg/kg b.w.).

### Postmortem examinations

A macroscopic examination of the implantation site was performed by observing the appearance of the tissue in situ and by photographic documentation. Any abnormalities were recorded with details of the location, color, shape and size. Two smears from the synovial fluid were prepared, stained using hematoxylin and eosin (H & E) and May-Grünwald-Giemsa and evaluated following the suggestions from the ISO guideline 10993–6, Annex E, Table E.1. From each implanted knee, the implantation site (femur cartilage at knee joint) was collected for H & E and Safranin-O staining. A histopathological evaluation of the administration site was performed using O’Driscoll scoring.

### O’Driscoll score

For the O’Driscoll score, range from 0 to 24 is used, with 24 as the best scoring indicator. It uses different characteristics to build the score including cellular morphology, Safranin-O staining of the matrix, surface regularity and structural integrity. Also the thickness of the cartilage in the healing zone, bonding to the adjacent cartilage, hypocellularity within the tissue, chondrocyte clustering and freedom from degenerative changes in adjacent cartilage are considered (Rutgers et al. 2010).

### Brittberg score

The Brittberg score is a macroscopic scoring system which evaluates the quality of defect repair tissue and the integration with the surrounding cartilage, as well as the macroscopic appearance. Each section can score 0–4 points, and so the overall score ranges from 0 to 12 points, with 12 points indicating the best result (Grade I, normal repair). Grade II (nearly normal) ranges from 8 to 11 points, Grade III (abnormal) from 4 to 7 points and Grade IV (severely abnormal) from 1 to 3 points (Brittberg 2000).

### Synovia smear

For the histopathological evaluation of the synovia smear, synovial cells and non-synovial cells (polymorphonuclear cells, lymphocytes, plasma cells, macrophages and giant cells) were included in the scoring system. Different gradings were divided into 5 groups with 0 as no cells, 1 as rare cells (1–5 cells in the evaluated segment), 2 as 5–10 cells, 3 as strong infiltrations and 4 as packed.

### Statistical analysis

A statistical analysis of data was performed for each group separately. Data values are reported as the mean ± standard deviation. Statistical analysis was performed using nonparametric Mann–Whitney U test with a confidence level of 95% or more.

## Results

### Postoperatively observations

Between the animal delivery and the surgery, the animals displayed a stagnating or positive body weight gain except for one rabbit, with a decrease in body weight as a result of reduced food consumption. In both treatment groups, single animals demonstrated a relieving posture of the right hind leg or an edema or hematoma formation, which was not observed in untreated animals. In 4 (out of 6) animals treated with 1.75% hyaluronan thiomer gel (HA gel), an increased incidence of swelling was noted at the surgery site only at some time points and in some animals with an increased body temperature (39.5 °C) and reduced food consumption. In comparison, the 1.75% hyaluronan thiomer gel and 1.5% hyaluronan thiomer matrix group (HA gel + matrix) exhibited a swelling at the surgery site in the first 3 weeks with reduced food consumption and discolored urine in 2 (out of 6) animals. A treatment-related influence could not be ruled out, but also the half of the untreated animals showed reddening or swelling of the skin in the surgery area. Nevertheless, all implantation sites and all animals were carried forward without any findings at the end of the second observation period.

### Macroscopic findings (Brittberg score)

No treatment-related findings were noted at the implantation site of any animals at either time point (Fig. 2a–c). At the end of the first observation period (4 weeks), a single nodule of soft consistency in subcutis near the implantation site was noted in 2 (out of 3) untreated animals. Also in 1 (out of 3) animals treated with HA gel and 2 (out of 3) animals treated with the HA gel + matrix. These findings could be the outcome from surgical injury and not influenced by the treatment method. At the end of the second observation period (12 weeks), the only macroscopic finding noted in one animal treated with HA gel was a red fluid in the uterus. The reason for this could not be clarified but was assumed to be unrelated to the test item.

**Fig. 1:**
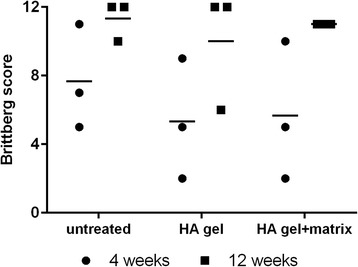
Macroscopic repair score of the regenerated tissue. Individual scores are represented as *dots* (4 weeks) and *squares* (12 weeks) with the arithmetic means of the untreated and treated groups shown as *horizontal bars*. The scores (1–12) represent the overall repair by assessing the initially grafted surface, integration to border zone and macroscopic appearance. No significant (n.s.) difference within a group or between the three groups is given in the two timepoints

The scores of macroscopic findings (Brittberg score, Fig. 1) varied considerably during the first observation time point (4 weeks after the implantation). 1 (out of 3) animals of each group showed a nearly normal regeneration with overall repair assessment grade. The remaining two animals in each group demonstrated an abnormal (grade III) to severely abnormal (grade IV) repair and regeneration. At the necroscopy time point 12 weeks after the implantation, 2 (out of 3) animals from the untreated group (empty defect) were observed with normal regeneration (grade I). The third animal was observed with nearly normal regeneration (grade II). Similarly, in the HA gel group, 2 (out of 3) animals demonstrated a normal regeneration process (grade I), while in one rabbit abnormal regeneration (grade III) was noted. All animals in the HA gel + matrix group were observed with nearly normal regeneration (grade II). In 2 (out of 3) animals, the regenerated tissue was above the level of the surrounding cartilage which could have resulted in a permanent mechanical irritation against the patella at a later time point if the recovery for these animals had been longer.

The results of the 12 week observation period were more homogenous, and a nearly normal regeneration was visible in all animals except one in the HA gel group.

### Histopathological findings (O’Driscoll score)

**Fig. 2:**
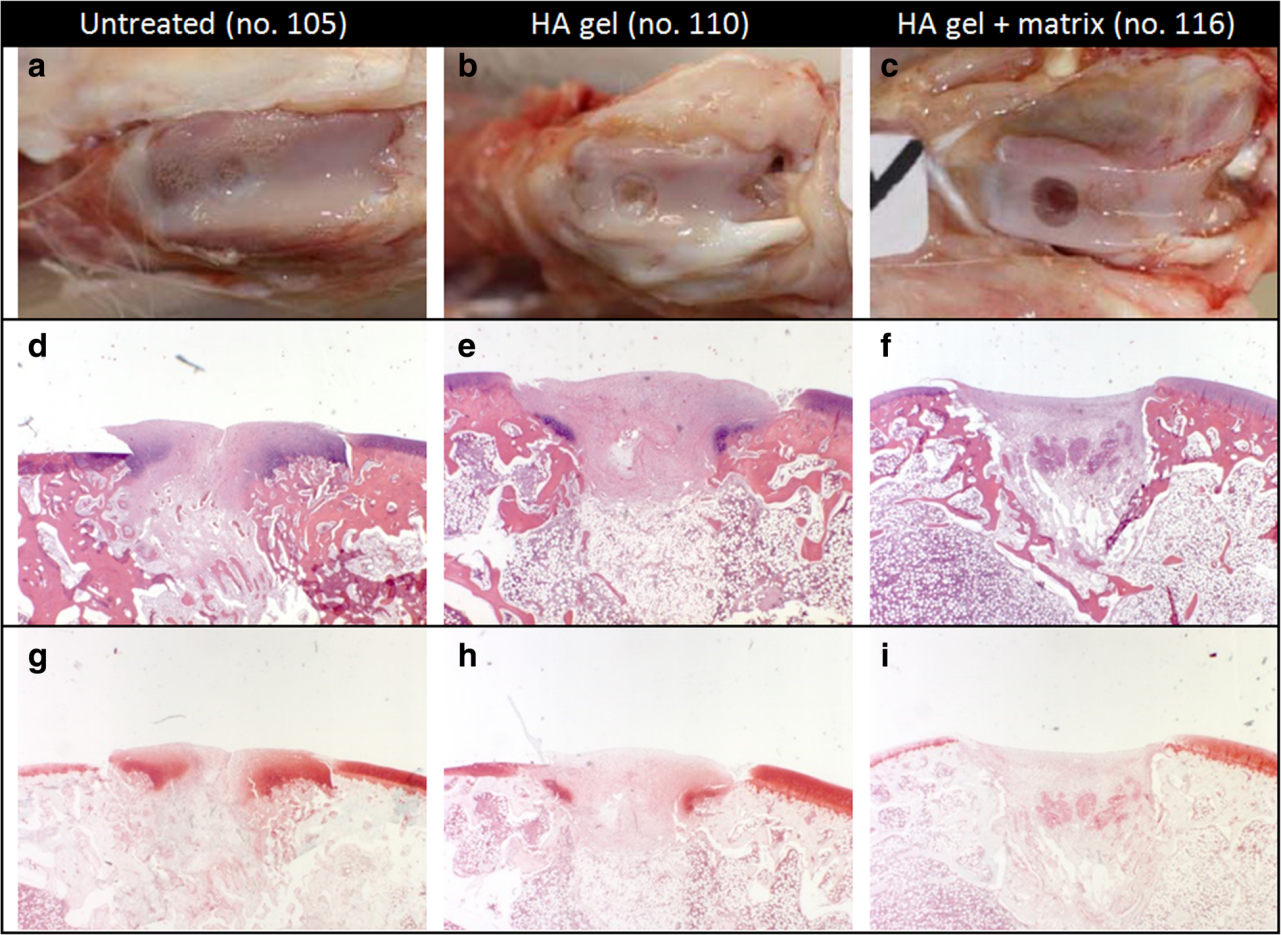
Gross morphology and histology of one representative rabbit per group sacrificed 4 weeks after treatment. **a**–**c** shows the implantation site of the trochlea of the femur. **d**–**f** represents cross-section histology of the implantation site stained with hematoxylin and eosin (H & E). In the untreated group (**d**) the defect was filled up with repair tissue, while in the treated ones the HA gel (**e**) or HA gel + matrix (**f**) is shown very well. **g**–**i** shows cross-section histology of the implantation site stained with safranin-O. In the untreated group (g) the highest proteoglycan staining (*red*) was detected. The HA gel group (**h**) shows a weak proteoglycan staining, while in the HA gel + matrix group (**i**) no proteoglycan was detected on the implantation site

The implantation sites of untreated animals reached a mean O’Driscoll score of 18.7 (maximum score 24) at the end of the 4 week observation time point (Fig. 2d–i). The average score was nearly comparable to the HA gel + matrix group (16.3) but was much higher than in the HA gel treated group (9.7). The reduction was mainly due to the absence of hyaline cartilage and proteoglycan. The inter-individual variation was high in the untreated and HA gel + matrix group, and lower in the HA gel treated group.

**Fig. 3:**
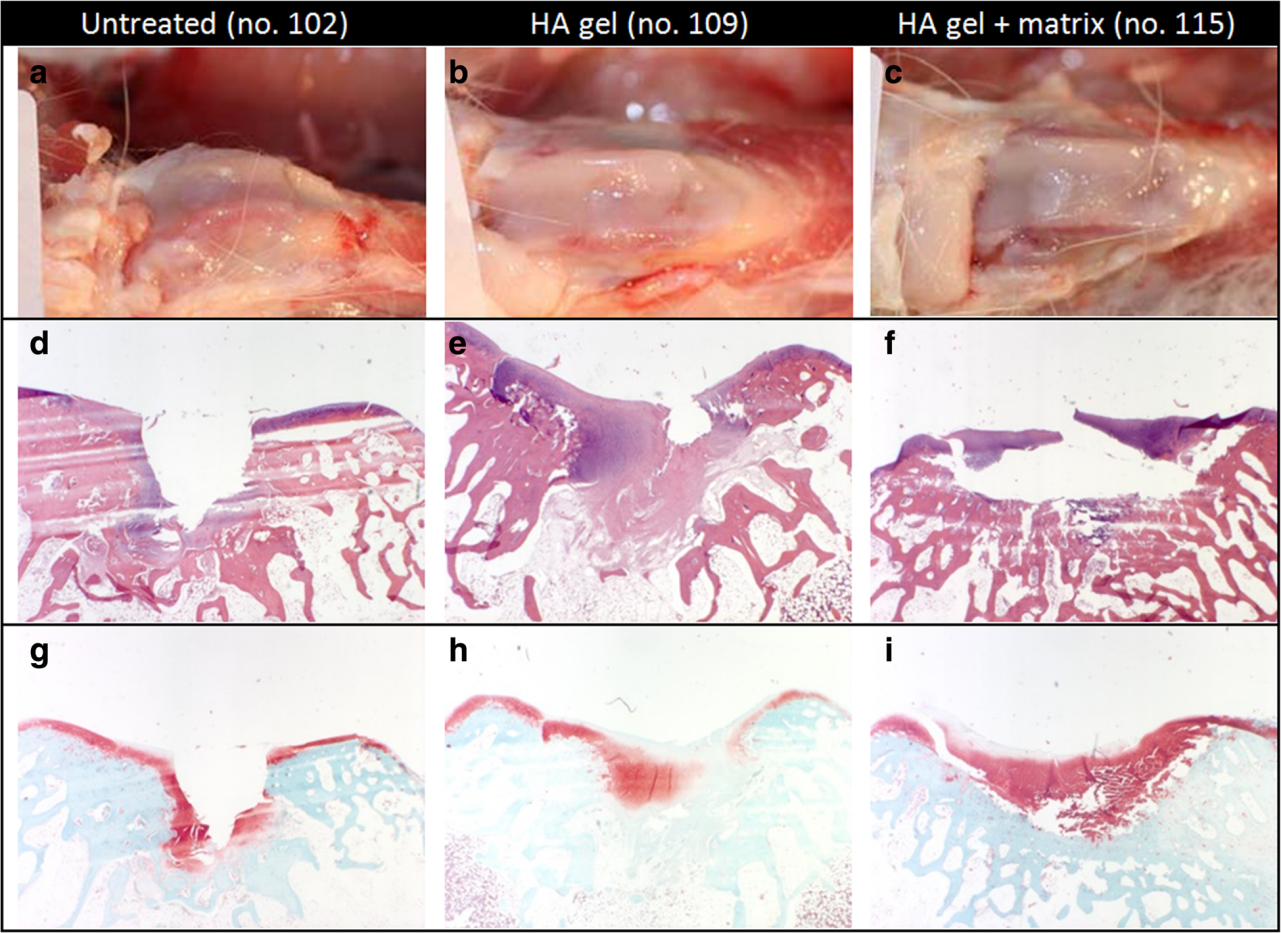
Gross morphology and histology of one representative rabbit per group sacrificed 12 weeks after treatment. a–**c** shows the implantation site of the trochlea of the femur. **d**–**f** represents cross-section histology of the implantation site stained with hematoxylin and eosin (H & E). In the untreated group (**d**) the defect was observed with little tissue formation, while the HA gel (**e**) or HA gel + matrix (**f**) enhanced better repair tissue with adequate cell distribution (*violet*). **g**–**i** shows cross-section histology of the implantation site stained with safranin-O. In the untreated group (**g**) a high proteoglycan staining (*red*) was detected in the almost existing defect. The HA gel group (**h**) shows more proteoglycan staining, but not as much observed in the group treated with HA gel + matrix (**i**)

**Fig. 4:**
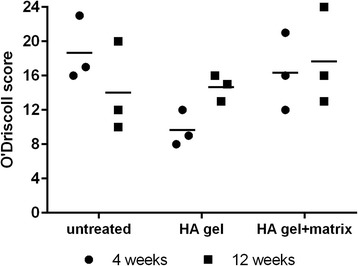
Cartilage repair score of the regenerated tissue. Individual scores are represented as *dots* (4 weeks) and *squares* (12 weeks) with the arithmetic mean of the untreated and treated groups shown as *horizontal bars*. The scores (maximum 24) represent the quality of the repaired cartilage tissue and is characterized by cellular morphology, safranin-O staining, surface regularity, structural integrity, thickness, bonding to the adjacent cartilage, hypocellularity, chondrocyte clustering and freedom from degenerative changes in adjacent cartilage. No significant (n.s.) difference within a group or between the three groups is given in the two timepoints

At the end of the 12 week observation period (Fig. 3d–i), the mean score of the untreated group (14.0) was comparable to the HA gel group (14.7) but slightly decreased in comparison to the HA gel + matrix group (17.7). About the first observation time point, a high inter-individual variation was seen after 12 weeks in the untreated or HA gel + matrix group than in the HA gel treated animals (Fig. 4).

**Fig. 5:**
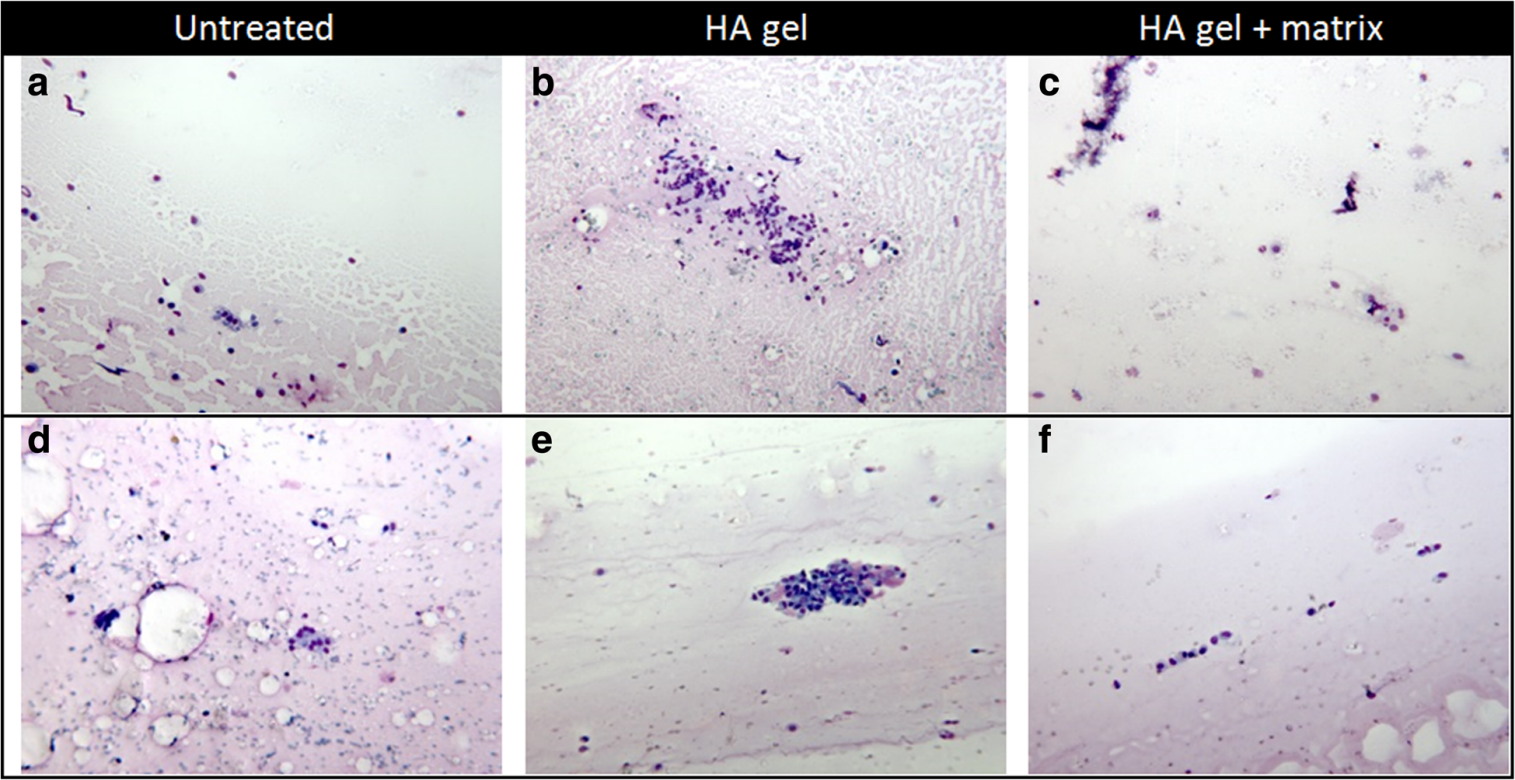
Histopathological evaluation of the synovial fluid smear. **a**–**c** shows the animal no. 105, 110 and 116 after 4 weeks of treatment and **d**–**f** shows the animal no. 102, 109 and 115 after 12 weeks of treatment as representative samples

Four weeks after implantation the synovial smear showed synovial cells (grade 1) in the untreated animals. In the HA gel treated group a mean score of 1 or 2 regarding the synovial cells could be shown, while in the HA gel + matrix animals non-synovial cells were determined after 4 weeks (Fig. 5a–c). At the end of the 12 week observation period, the untreated group showed a higher infiltration of synovial cells (grade 2 to 3) than other cells (grade 1 to 2). In both treated groups a score of 0–2 was evaluated for synovial cells as well as non-synovial cells (Fig. 5d–f).

## Discussion

The main findings of this study in a surgically induced chondral defect of NZW rabbits were: 1) macroscopic observations revealed no differences regarding inflammatory response when the defect was filled with either a hyaluronan thiomer gel (HA gel) or hyaluronan thiomer gel + hyaluronan thiomer matrix (HA gel + matrix) at the transplantation site. 2) Histopathological results denoted that the HA gel + matrix group enhanced comparable cartilage tissue repair at the defect with an increase in cell growth around the tissue after 4 weeks compared to HA gel alone and the empty defect control. The tissue repair process was delayed in the first week’s post implantation of HA gel alone group into the defect; however, the tissue repair process was similar to HA gel + matrix group as well as the empty defect control at the end point of evaluation. Together we observed that the tissue repair process is inconsequential to either of these treatment groups post 12 weeks.

The results are in accordance with reported studies that have explicated that an injectable gel or matrices of hyaluronan may have a healing potential in articular cartilage defects (Cecilia et al. 2010; Leela et al. 2008). Cross-linked hyaluronan gels or matrices by chemical modification not only provide mechanical stability but also retain the biocompatibility and a slower retention of degradation (Collins and Birkinshaw 2008). Although the surgical defect in all groups was induced in the trochlear groove which is primarily secluded from the synovium and provided with a prominent blood supply, no immune response was witnessed upon implantation.

It has been demonstrated in a rabbit model, that an adaptive immune response in defects from the patellar and trochlear groove upon implantation of an allogenic matrix is primarily intermediated by the vicinity of the synovium and not in contact with the blood supply (Arzi et al. 2015). The clinical evaluation revealed that an edema or a relieving posture of the hind leg at the defect site in the HA gel or HA gel + matrix groups, followed by an increase in the incidence of swelling, increased body temperature, reduced food consumption and discolored urine or feces in the test subjects. Nevertheless, the body weight gain was not influenced post implantation.

Limitations to our current study include the 4 mm^2^ lesion size criteria induced in the chondral defect. A larger defect could have prevented the endogenous healing in the empty defect control group. Another limitation of our study is the endogenous healing capacity in rabbit knees which is a well-known occurring phenomenon (Nishizawa et al. 2010; Terajima et al. 2012; Kawamura et al. 1998). Wei et al. earlier reported when untreated full-thickness cartilage defects were studied in young, adolescence and adult rabbits; spontaneous tissue repair occurred with a faster healing in young animals than adults despite the compromise of mechanical stiffness than that in the native tissue (Wei and Messner 1999). This study emphasized the potential of self-healing in rabbits decrease over age and maturation of the tissue. In our present study, the rabbits investigated were 8-month-old young animals reporting the same mechanism of self-healing in the untreated defect control groups. This marks the evaluation among other treatment groups for a less comparative analysis to the untreated defect denoting the rabbit model relatively less reliable to our study design.

## Conclusions

Our study implies cartilaginous tissue formation in vivo by using a cross-linked hyaluronan as a provisional scaffold. However, the rabbit animal model in the current study resulted in a spontaneous tissue repair in the untreated control group. It is of importance to consider a relevant in vivo model to develop further proof of concepts from an in vitro study to a preclinical model. In summary, we have tested a cytocompatible space filing HA gel and gel + matrix that fills in space around defects to cartilage in situ. The HA gel alone or gel + matrix combination is a significant carrier for an arthroscopically matrix-assisted cartilage repair technique for cell or cell-free regenerative therapies. The in situ defect filling carrier referred in here can support in vitro and in vivo matrix formation, ensuring its stability in articular cartilage defects in vivo for up to 12 weeks.
